# GOLFIG Chemo-Immunotherapy in Metastatic Colorectal Cancer Patients. A Critical Review on a Long-Lasting Follow-Up

**DOI:** 10.3389/fonc.2019.01102

**Published:** 2019-11-08

**Authors:** Michele Caraglia, Pierpaolo Correale, Rocco Giannicola, Nicoletta Staropoli, Cirino Botta, Pierpaolo Pastina, Antonello Nesci, Nadia Caporlingua, Edoardo Francini, Laura Ridolfi, Enrico Mini, Giandomenico Roviello, Domenico Ciliberto, Rita Maria Agostino, Alessandra Strangio, Domenico Azzarello, Valerio Nardone, Antonella Falzea, Salvatore Cappabianca, Marco Bocchetti, Graziella D'Arrigo, Giovanni Tripepi, Pierfrancesco Tassone, Raffaele Addeo, Antonio Giordano, Luigi Pirtoli, Guido Francini, Pierosandro Tagliaferri

**Affiliations:** ^1^Department of Precision Medicine, University of Campania “L. Vanvitelli”, Naples, Italy; ^2^Biogem Scarl, Institute of Genetic Research, Laboratory of Precision and Molecular Oncology, Ariano Irpino, Italy; ^3^Medical Oncology Unit, “Bianchi-Melacrino-Morelli” Grand Metropolitan Hospital, Reggio Calabria, Italy; ^4^Medical Oncology Unit, Department of Experimental and Clinical Medicine, Magna Graecia University, Catanzaro, Italy; ^5^Radiation Oncology Unit, Siena University Hospital, Siena, Italy; ^6^Unit of Pharmacy, Section of Anti-blastic Drugs, “Bianchi-Melacrino-Morelli” Grand Metropolitan Hospital, Reggio Calabria, Italy; ^7^Medical Oncology Unit, Siena University Hospital, Siena, Italy; ^8^Immunotherapy, Cell Therapy and Biobank, Istituto Scientifico Romagnolo per lo Studio e la Cura dei Tumori IRCCS, Meldola, Italy; ^9^Section of Clinical Pharmacology and Oncology, Department of Health Sciences, School of Medicine/Translational Oncology Unit, Careggi University Hospital, University of Florence, Florence, Italy; ^10^Statistical Unit, IFC-CNR (CNR), Grand Metropolitan Hospital-IFC, Reggio Calabria, Italy; ^11^Oncology Unit, Day Hospital, San Giovanni di Dio Hospital, ASL Napoles 2 Nord, Frattamaggiore, Italy; ^12^Sbarro Institute for Cancer Research and Molecular Medicine and Center of Biotechnology, College of Science and Technology, Temple University, Philadelphia, PA, United States; ^13^Department of Medical Biotechnology, University of Siena, Siena, Italy

**Keywords:** colorectal cancer, metastatic, chemotherapy, immunotherapy, GOLFIG, phase III clinical trial, real-world medicine

## Abstract

**Background:** GOLFIG is a chemo-immunotherapy regimen established in preclinical models that combines gemcitabine + FOLFOX (fluoropyrimidine backbone coupled to oxaliplatin) poly-chemotherapy with low-dose s. c. recombinant interleukin-2 (rIL-2) and granulocyte-macrophage colony stimulating factor (GM-CSF). Promising antitumor effects in metastatic colorectal cancer (mCRC) patients were obtained in previous phase II and III trials. Here we report the results of 15 years of follow-up.

**Methods:** This is a multi-institutional retrospective analysis including 179 mCRC patients receiving GOLFIG regimen between June 2002 and June 2018. Sixty-two of them received the treatment as frontline (enrolled in the GOLFIG-2 phase III trial) and 117 as second/third line (49 enrolled in the GOLFIG-1 phase II trial and 68 as compassionate use). One hundred twelve patients showed a primary left side and 67 a primary right side; K/N-ras mutational status was available in 74 cases, and an activating mutation was detected in 33. Kaplan–Meier and Cox regression analyses were carried out to relate PFS and OS with different parameters.

**Results:** Overall, we recorded a mean PFS and OS of 15.28 (95% CI: 10.36–20.20) and 24.6 (95% CI: 19.07–30.14) months, respectively, with 14 patients surviving free of progression for 10 years. This regimen, in our updated survey of the GOLFIG-2 trial, confirmed superiority over FOLFOX in terms of PFS (hazard ratio (HR) = 0.58, *p* = 0.006) with a trend to a longer OS (HR = 0.69, *P* = 0.06) in the first line. Our analysis also confirmed significant antitumor activity in pre-treated patients, reporting a mean PFS and OS of 12.55 (95% CI: 7.19–17.9) and 20.28 (95% CI: 14.4–26.13) months, respectively. Immune-related adverse events (irAEs) were recorded in 24% of the cases and were related to a longer survival (HR = 0.36; *P* = 0.0001). Finally, patients' outcome was not correlated to sex, sidedness, and MT-K/N-ras.

**Conclusions:** The GOLFIG regimen is a reliable underestimated therapeutic option in pre-treated mCRC patients and offers a strong rationale to design further trials.

## Introduction

Colorectal carcinoma (CRC) is one of the leading causes of cancer-related deaths worldwide (1). At the present, first- and second-line treatment of metastatic CRC (mCRC) has been well-defined and is based on the use of a fluoropyrimidine backbone [fluorouracil (5-FU) ± capecitabine or levofolinate (LF)] coupled to oxaliplatin (FOLFOX/CapeOX), irinotecan (FOLFIRI/CapeIRI), or even both (FOLFOXIRI). Monoclonal antibody (mAB) therapy may also be associated, for example, with bevacizumab, against VEGF (vascular-endothelial growth factor) (2, 3), or with panitumumab and cetuximab, against EGFR (epidermal growth factor receptor) (3, 4) (only applicable if patients are not expressing mutations that activate K/N-ras) (5). An alternative use of these regimens is recommended in the second line with no real difference in terms of outcome, with the only exception of the regimen of FOLFIRI and aflibercept, an anti-angiogenetic recombinant protein able to trap VEGF A/B and the placental growth factor (PlGF), which showed an advantage over chemotherapy alone in terms of response rate (RR) (19.8 vs. 11.1%; *P* = 0.0001), PFS [6.90 vs. 4.67 months; hazard ratio (HR) = 0.758; IC95% 0.661–0.869; *P* < 0.0001], and OS (13.50 vs. 12.06 months; HR = 0.817; IC34% 0.713–0.937; *P* = 0.032). The latter regimen, however, is reserved for fit patients since it is associated with potentially severe adverse events including bleeding, hypertension, infections, and gastro-enteric and hematological toxicity in almost 30% of the patients who refuse to continue the treatment (6, 7). Almost half of mCRC patients over second-line disease progression are still fit to receive further treatments with regorafenib or trifluridine/tipiracil. The first one is a multi-kinase inhibitor with potent anti-angiogenetic and cytostatic effects, while the second is a DNA-damaging cytotoxic pro-drug. Both of them, investigated in two multi-institutional phase III trials (CORRECT and RECOURSE trials) in pre-treated mCRC patients, reported similar advantage over best supportive care (BSC) in terms of PFS (2 vs. 1.7; *p* < 0.001) and OS (6–7 vs. 5 months; *P* < 0.01) but with severe and drug-specific adverse events and costs (8–10). Overall, the survival of mCRC patients remains in the range of 26–28 months, with no real improvement achieved in the last 10 years. On these bases, research on new and more active treatment strategies is strongly needed.

In the last few years, the interest in the use of immunological anticancer strategies is greatly increased due to the clinical development of PD-1/PDL-1 immune-checkpoint blockade with mABs (11, 12). Although very active in the treatment of aggressive and heterogeneous malignancies such as NSCLC, malignant melanoma, and head and neck and esophageal cancer, these strategies resulted as inactive in mCRC patients not bearing specific deficit in the mismatch repair complex and microsatellite instability, usually expressed in <5% of cases (13–15).

Many different immunological strategies, including immune-modulating agents, mAbs, cytokines, and cancer vaccines, in mCRC patients have been evaluated in the last 25 years, with contrasting results in terms of clinical efficacy. Even though they failed to demonstrate a clear antitumor effect, these studies produced a large amount of data concerning the ability of different immune-modulating agents to trigger an efficient tumor-specific adaptive immune response, to activate mechanisms of immune resistance and to produce immune-related adverse events (irAEs) (16–25).

On the track of those studies, we demonstrated the possibility of eliciting highly efficient colon cancer–specific cytotoxic T-cell lines (CTLs) by *in vitro* stimulating human peripheral blood mononuclear cells (PBMCs) with colon cancer cells pre-exposed to immunomodulating drugs including gemcitabine, oxaliplatin, LF, and fluorouracil (5-FU) alone or in combination (GOLF) and other chemo-immunological blends, such as granulocyte-macrophage colony stimulating factor (GM-CSF) and then low-dose human recombinant interleukin-2 (IL-2) (26, 27). GM-CSF was used to activate the antigen-presenting ability of the dendritic cells (DCs) expressed in human PBMCs (0.5–2% of the whole population), while IL-2 was required to promote the proliferation of cross-primed CTL clones (26, 27). In this context, the GOLF multidrug combination showed the unique ability (not shared with FOLFOX or FOLFIRI) to induce a massive release of antigenic material from tumor cells also activating a strong immune-danger signal able to empower the subsequent DC-mediated cross-priming, leading to the generation of CTL precursors with enhanced antitumor activity *in vitro* (26–28).

These results offered the rationale to design an innovative treatment chemo-immunotherapy regimen aimed to mimic the abovementioned protocol for the *in vitro* sensitization of human colon cancer–specific CTLs. We combined a biweekly chemotherapy with gemcitabine + FOLFOX-4 (GOLF regimen) (administered on days 1–2q15) integrated by subcutaneous (sc.) administration of GM-CSF (sargramostim/molgramostim) to activate peripheral DCs (days 3–7), followed by bi-daily sc. administration of recombinant IL-2 (aldesleukin) at a very low dose (days 8–14). (29–31). The combination of GOLF poly-chemotherapy with GM-CSF and IL-2, designated as GOLFIG, was evaluated by two subsequent multicenter phase II and phase III clinical trials in mCRC patients. The GOLFIG-1 trial, started in 2002, enrolled 46 mCRC consecutive patients who received prior chemotherapy with fluoropyrimidine alone (12 cases), FOLFOX-4 chemotherapy alone (24), or both FOLFOX and FOLFIRI (10 cases). It resulted as moderately safe and showed significant immunological and antitumor activity. The study reported a response rate (RR) and a disease control rate (DCR) of 56.5 and 91.3%, respectively, and a mean PFS of 12.3 months (30, 31).

In these patients, to confirm its biological rationale, the study was accompanied by ancillary immune monitoring, which recorded a progressive treatment-related increase in CTL precursors specific for tumor-associated antigens such as carcino-embryonic antigen (CEA) and thymidylate synthase (TS); an increase of central memory-cytotoxic T-lymphocytes (CD8+CD45RA-CCR7+) (Tcm), activated CTLs (CD8+CD62L+), and highly cytotoxic NK (CD3-CD56+CD16+) cells; and a parallel decline in immunosuppressive regulatory T-cells (CD4+CD25hi+FoxP3+) (Tregs). These events were recorded both in patients' PBMCs and in the tumor tissue on a sample of patients who underwent a post-treatment biopsy or palliative surgery (30, 31).

In this first trial, we reported for the first time the evidence of self-limiting irAEs in 19% of cases, whose appearance was highly correlated to a favorable outcome (31–33). Altogether, those results granted the rationale to design a subsequent multicenter phase III trial where mCRC patients were randomized to receive up front the GOLFIG regimen or FOLFOX-4 regimen, which, at the time of the protocol design (2004), was considered as the best frontline treatment for the advanced disease. The trial enrolled 124 patients (62 per arm) before being prematurely terminated due to the sudden withdrawal of salgramostim from the European market and due to delayed recruitment related to the rise of many competing phase III trials investigating FOLFOX/FOLFIRI chemotherapy in addition to mAbs, like bevacizumab, cetuximab, and then panitumumab. The results of the first interim analysis of the GOLFIG-2 trial were obtained on the first 98 censored patients (49 per arm), with a median follow-up of 5 years. Our data indicated a clear advantage of GOLFIG chemo-immunotherapy over the FOLFOX regimen in terms of PFS (18.26 vs. 7.82 months, HR = 0.52, *P* < 0.002), RR, and DCR, fulfilling the primary objective of the study. In the experimental arm of the trial, we recorded irAEs in 12% of the cases, which was strongly correlated with a very positive outcome in terms of PFS (HR: 0.48; *P* = 0.031) and survival (HR: 0.42; *P* = 0.028). The immune monitoring of the patients enrolled in this second trial confirmed the same immunological findings reported in the GOLFIG-1 only in the experimental arm, while minimal effects were recorded in patients receiving FOLFOX chemotherapy alone (34). These studies did not have further experimental developments, due to the establishment of very effective multidrug combinations with mAbs to EGFR and VEGF, which led to the definition of solid guidelines for the first and second treatment lines of mCRC patients. In light of the positive premises, the GOLFIG chemo-immunological treatment could still be used as compassionate use in a cohort of 68 mCRC patients who had progressed through the standard sequence of treatment and still presented an acceptable performance status and wish for treatment.

On these bases, we decided to carry out a retrospective analysis including all the mCRC patients who have received the GOLFIG chemo-immunotherapy between June 2002 and December 2018.

## Patients and Methods

### Study Design and Participants

This is a multi-institutional retrospective analysis assessed on a cohort of 179 patients with mCRC who had received GOLFIG chemo-immunotherapy between June 2002 and June 2018. Our database included patients enrolled in the GOLFIG-1 phase II trial (49 cases), GOLFIG-2 phase III trial (EUDRACT: 2005-003458-81) (62 cases enrolled in the experimental arm and 62 who received standard FOLFOX chemotherapy in the control arm) (31, 34), and compassionate real-world therapy (68 cases) after multiple treatment lines with FOLFOX/FOLFIRI chemotherapy +/– cetuximab or bevacizumab (Table 1). All the patients received the treatment at MOU-RC, MOU-SI, MOU-CZ, ROU-SI, TOU-FI, and ICT-FL.

**Table 1:** Clinical features of 179 mCRC patients who received GOLFIG chemo-immunotherapy between June 2002 and June 2018.

**Sex**	**n**.	**Treatment line**	**n**.	**Primary sidedness**	**n**.	**K/N-ras**	**n**.	**irAEs**	**n**.
Male	106	First line	62	Left	112	WT	41	No	136
Female	73	> 2 lines	117	Right	67	Mut	33	Yes	43

*Sixty two of them had been enrolled in the GOLFIG-2 phase III trial and received frontline treatment. One hundred seventeen patients received salvage treatment after two/three treatment lines. Forty-nine of them had been enrolled in the GOLFIG-1 phase II trial, and 68 received the GOLFIG chemo-immunotherapy as real-world treatment. mCRC, metastatic colorectal cancer; GOLFIG, gemcitabine, oxaliplatin, LF, and fluorouracil poly-chemotherapy with granulocyte-macrophage colony stimulating factor and interleukin-2); irAEs, immune-related adverse events*.

### Posology Schedule

Gemcitabine (1,000 mg/m2) and oxaliplatin (85 mg/m2) were administered to all patients twice a week, on days 1–5 and 2–6, respectively, together with 5-FU as a bolus (400 mg/m2) and as a 24 h infusion (800 mg/m2) on days 1, 2, 15, and 16 and levofolinate acid (100 mg/m2) on days 1, 2, 15, and 16. Moreover, on days 3 and 7, 100 ug of s.c. GM-CSF was administered, and on days 8–14 and 17–19, twice a day, an ultra-low-dose s.c. recombinant interleukin-2 (rIL-2) (0.5 × 106 IU) (34).

### Evaluation

The standard assessment was performed at baseline and every 4–6 weeks (clinical history, hemato-chemical analysis, physical examination, CA19.9 and CEA assays, ultrasound scans, and chest X-ray). Moreover, every 3 months, recorded high-definition, multi-slice computed tomography scans with contrast medium were recorded. According to the standard WHO criteria, OS, PFS, overall response, and adverse events assessed were evaluated. irAEs were also evaluated by an expert rheumatologist and mainly consisted of a cutaneous rash, polyarthralgia, and thyroiditis. There was one case of lupus discoides and one case of Sjogren's syndrome, both reported as case reports as long-lasting patients (32, 33).

Patients enrolled in the GOLFIG-1 phase II trial and real-word treatment received the treatment after one/two treatment lines. Conversely, those enrolled in the GOLFIG-2 phase III trial received the treatment as frontline upon randomization in two different arms. Patients in the control arm received standard treatment (according to guidelines defined in 2005) with the FOLFOX-4 regimen, while those in the experimental arm received the GOLFIG chemo-immunotherapy.

Criteria of inclusion were the mCRC confirmed diagnosis (histology) and signed informed consent. Moreover, patients had to show a normal renal, hepatic, and cardiac function; an ECOG performance status ≤ 2; and a blood composition not critically compromised (with more than 2,500 white blood cells per mm3, more than 96 g of hemoglobin per dL, and more than 100,000 platelets per mm3). Criteria of exclusion were: the presence of other malignancies, active infections, immunosuppression, and autoimmune major diseases. Moreover, central nervous system involvement and major organ failure were criteria for exclusion. The study was approved by bioethical committees of the different institutions and designed according to good clinical practice recommendations.

During the GOLFIG-2 trial randomization and masking, computer-generated patients' randomization was carried out at MOU-SI, and each recruiting center communicated with patients by telephone/fax. An equal number of patients were randomly assigned to the control arm (FOLFOX-4) or to the experimental arm (GOLFIG). The allocation of the treatment was unmasked (34). We have programmed to continue to enroll patients in the future, administering the regimen in compassionate use according to the availability of both GM-CSF and r-IL2.

### Statistical Analysis

Our database, including these data, was used for our real-world statistical analysis with a median follow-up of 15 years. We used the Kaplan–Meier method to carry out descriptive analyses. We estimated HR with Cox regression analysis and compared OS and PFS between treatment groups with the log-rank test. Using the median values as a cutoff, we then transformed continuous variables into categorical variables and performed a *post-hoc* subgroup analysis of OS and PFS for the evaluation of characteristics of the different baselines of patients. We constructed a forest plot to report the significance level of single interactions, HR, and 95% CI. We used the w2 and Student *T*-tests according to the continuous or dichotomic nature of the variables to investigate their association. Moreover, to confirm the role of significant variables in patients' outcome, we used the Cox proportional hazard model. We analyzed the data using SPSS and GraphPad Prism 3.2 software.

## Results

### Patient Population and Stratification

Our analysis included 179 mCRC patients. Sixty-two patients who received the GOLFIG regimen as frontline treatment had been enrolled in the GOLFIG-2 phase III trial. In that study, an equivalent control cohort of 62 patients randomized to receive the FOLFOX-4 regimen was also enrolled (34). Further, 117 cases evaluated in the current study received the GOLFIG regimen upon progression over multiple treatment lines (Table 1). Concerning the last group of patients, 49 cases had been enrolled in the GOLFIG-1 phase II trial (30, 31), while a further 68 received the treatment as compassionate use after they had received FOLFOX and FOLFIRI +/– mAbs to VEGF or EGFR as reported in Table 2.

**Table 2:** Clinical features of 68 mCRC patients who received the GOLFIG regimen on compassionate use between August 2008 and June 2018.

	**Sex**		**ECOG**		**Prev. Cht. lines**		**Prev. mAbs to**				**Radiotherapy**		**Sidedness**		**k/n-ras**		
	***Male***	***Female***	***≤1***	***>1***	***1***	***≥2***	***None***	***EGFR***	***VEGF***	***Both***	***Yes***	***Not***	***Left***	***Right***	***WT***	***M“t***	***ND***
N	40	28	52	16	8	60	11	24	14	19	14	54	46	22	46	18	4
%	58.8	41.2	76.5	23.5	11.8	88.2	16.2	35.3	20.6	27.9	20.6	79.4	67.7	32.3	67.6	26.5	5.9

Overall, were included 106 males and 73 females, with a median age of 64 years and a median follow-up of 120 months, with PS ECOG 0–2. The tumor was derived from the left colon side in 112 patients and from the right side in the remaining 67 cases. K/N-ras mutational status was available for 74 pts, with 33 of them showing an activating K/N-ras mutation (Tables 1, 2).

### Toxicity

The regimen proved to be quite safe in both chemo-naïve and pre-treated patients. Grade 1–2 fever during sc. aldesleukin administration was the most frequent adverse event (39 patients; 19.5%). Low-grade (grade 1–2) asthenia/fatigue, anemia, neutropenia, thrombocytopenia, and gastro-enteric toxicity were also recorded in 10–15% of the cases. In 6, 7, and 10 cases of pre-treated patients group, gastro-enteric toxicity, thrombocytopenia, and grade 3–4 anemia episodes (reversible) were reported, respectively. Twelve patients showed hypersensitivity to oxaliplatin with hypotension, erythema, dyspnea, and fever, while GM-CSF–related bone pain and oxaliplatin-related neurologic toxicity were also recorded to lesser extents (2–5%). After 4–9 months from the beginning of the treatment, self-limiting irAEs were recorded in 49 patients (24%), mainly consisting of mono/oligo-articular arthritis. Discoid lupus erythematosus and Sjogren's syndrome were also described in two patients (32, 33). These events were all self-limiting and/or controlled with non-steroidal anti-inflammatory drugs (31, 34). There was no statistical difference in irAE frequency comparing chemo-na**ï**ve with pre-treated patients (*P* = 0.49).

### Antitumor Activity

**Figure 1:**
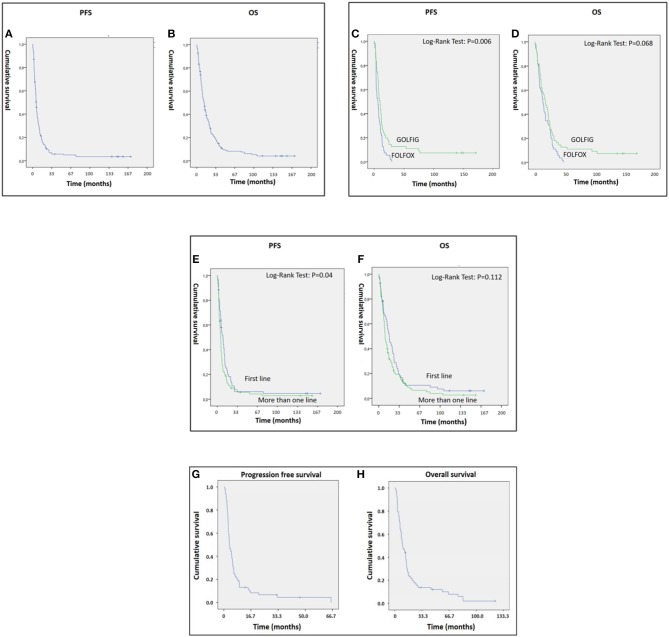
**(A–H)** Panels A and B represent PFS **(A)** and OS **(B)** of metastatic colorectal cancer (mCRC) patients who received the GOLFIG (gemcitabine, oxaliplatin, LF, and fluorouracil poly-chemotherapy with granulocyte-macrophage colony stimulating factor and interleukin-2) regimen and enrolled in the GOLFIG-1 trial, GOLFIG-2 trial, and real-world treatment. **(C,D)** represent the updated results of the GOLFIG-2 trial including 124 mCRC patients randomized to receive frontline treatment with GOLFIG regimen (62 pts) or FOLFOX (fluoropyrimidine backbone coupled to oxaliplatin) chemotherapy (62 pts). GOLFIG showed superiority over FOLFOX in terms of PFS **(C)** and a trend to superiority in terms of OS **(D)**. All of the patients were allowed a free second line with or without anti–epidermal growth factor receptor (EGFR) or vascular-endothelial growth factor (VEGF) monoclonal antibodies (mABs). **(E,F)** represents the PFS **(E)** and OS **(F)** of mCRC patients who received GOLFIG chemo-immunotherapy as frontline (62 pts enrolled in the GOLFIG-2 trial) or second/third line (117 patients, of whom 49 enrolled in the GOLFIG-1 trial and the remaining as real-world treatment). **(G,H)** represent PFS **(G)** and OS **(H)** of the real-life subset of patients.

Overall, we found a mean PFS and OS of 15.28 [95% CI: 10.36–20.20] and 24.61 [95% CI: 19.07–30.14] months, respectively, with 14 patients who survived more than 10 years free of progression (Figures 1A,B). In the present study, we performed an updated analysis of the GOLFIG-2 trial in chemo-naïve mCRC including 62 censored patients with a 10-year follow-up, confirming that the GOLFIG regimen is superior to FOLFOX alone in terms of PFS [9.4 vs. 5.8 months; HR = 0.58 (CI 0.39–0.86), *P* = 0.006] and OS [17.3 vs. 12.9 months; HR = 0.69 (CI 0.47–1.03), *P* = 0.068] (Figures 1C,D).

These data confirmed the results of the previous analysis performed on 84 censored patients (42 patients per arm) with a much shorter follow-up (34).

**Figure 2:**
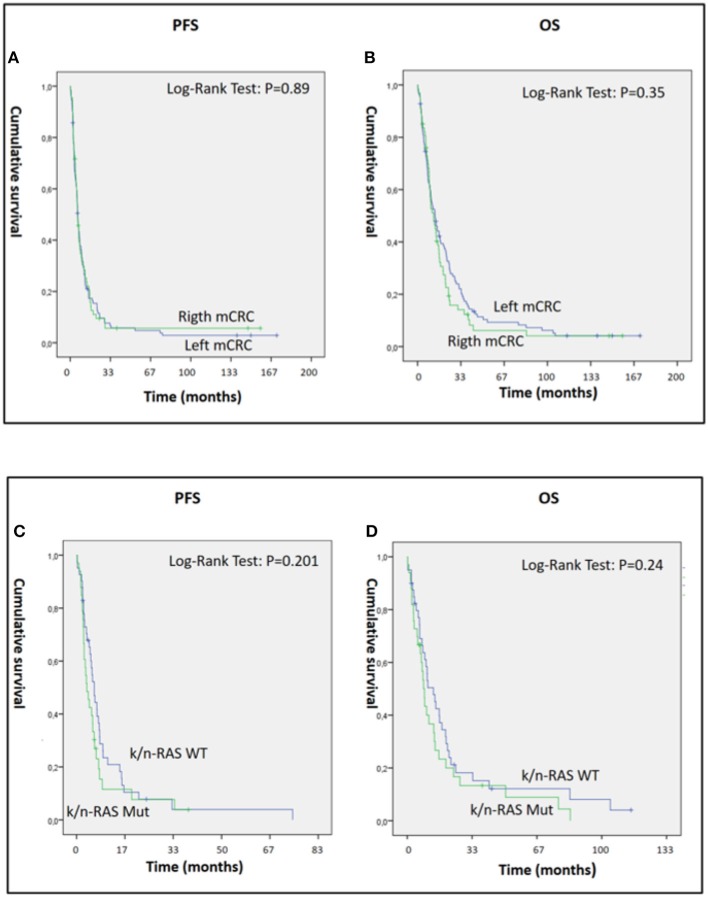
**(A–D)** Panels A and B represent PSF **(A)** and OS **(B)** of 179 mCRC patients who received GOLFIG chemo-immunotherapy with left (112 pts) and right primary sidedness (67 pts). **(C,D)** represent PFS **(C)** and OS **(D)** of 74 mCRC patients who received GOLFIG chemo-immunotherapy with wt (41 pts) and mutated K/N-ras (33 pts). Both primary tumor sidedness and K/N-ras mutational status did not influence the outcome of these patients.

The present study also demonstrated significant antitumor activity in largely pre-treated patients, reporting a mean PFS and OS of 12.55 [95% CI: 7.19–17.9] and 20.28 [95% CI: 14.4–26.13] months, respectively (Figures 1E,F). These results were of particular interest considering that 34 out 46 patients in the GOLFIG I trial had already received at least a previous chemotherapy line according to the FOLFOX regimen. Additionally, the majority of the 68 patients who received the GOLFIG treatment in the compassionate setting had already received multiple chemotherapy lines with FOLFOX and FOLFIRI regimens +/– mAbs to EGFR or VEGF (Table 2) according to the current guidelines. This real-life subset of patients showed a mean PFS and OS of 8.19 (4.8–11.5) and 19.19 (12.98–25.29) months, respectively (Figures 1G,H), which was perfectly in line with what was recorded in the original study. Further analyses were also carried out to investigate patients' outcome correlation with baseline neutrophil counts, irAEs, sidedness, mutational K/N-ras status, age, sex, performance status (ECOG ≤1), and multiple chemotherapy lines, finding that both PFS and OS were positively correlated with baseline neutrophil counts ≤ 4.500 cells/μl [HR: 0.32 (CI 0.21–0.45), *P* = 0.003] and occurrence of irAEs [HR = 0.36 (CI 0.25–0.53), *P* = 0.0001] only [Figures 2A–D and data not shown]. In fact, sidedness, mutational K/N-ras status, age, sex, and performance status (ECOG ≤1) did not correlate with both PFS and OS.

## Discussion

This study was performed to define the safety and antitumor activity of GOLFIG chemo-immunotherapy in mCRC patients on a long-lasting follow-up and also carried out an updated analysis on the GOLFIG-2 trial as frontline treatment of these patients (35). The present analysis of the GOLFIG-2 trial performed on a larger series of patients (124 vs. 98 cases evaluated in the last analysis in 2013) confirmed the superiority of GOLFIG chemo-immunotherapy over a FOLFOX-4 regimen as frontline treatment in terms of PFS, also reporting a trend to a prolonged survival in the GOLFIG arm (HR = 0.69; *P* = 0.06), with 7.8% of these patients and none in the FOLFOX arm alive and free of progression after 10 years, fulfilling the primary objective of the original study (34). These results are of interest considering that the study could also include symptomatic patients with a low performance status (ECOG≤2) who usually are excluded by the combined use of mAbs due to the high risk of toxicity (34), and that the trial allowed the possibility to use free second-line poly-chemotherapy in combination with anti-VEGF and anti-EGFR mAbs. Additionally, we should take into consideration that the most reliable frontline treatments at the present achieve an average RR of 34–55% and a PFS of 7–9 months, similar to what was reported in the GOLFIG arm but associated with more frequent adverse events and 10-times-higher costs. This evidence, although obtained on a small sample of patients, provides a strong rationale to continue the investigation on the GOLFIG regimen alone and in combination with anti-EGFR and VEGF mAbs.

Our current analysis was also performed on largely treated mCRC patients, including 49 patients enrolled in the GOLFIG-1 phase II trial (30, 31) and 68 treated on compassionate use after the progressed multiple treatment polylines alone or associated with anti-EGFR and VEGF mABs.

The GOLFIG-1 was a phase II trial designed on the clinical translation of preclinical immuno-oncological results (28–31) and started in 2002, long before either bevacizumab or cetuximab was available for the standard treatment of mCRC patients. The schedule was designed on the basis of the strong preclinical and clinical evidence that the addition of gemcitabine to oxaliplatin, 5-FU, and LF strongly potentiated both the direct anti-cancer effects and the immunological properties of the tumor. The results of that trial, including a small sample of patients, were encouraging in terms of safety, RR [56.5% (95% CI: 42.1–69.8)] and average time to progression (12.26, 95% CI: 9.2–15.2 months) (31). In the present analysis, we included an additional real-life sample of 68 mCRC patients who received the GOLFIG regimen on compassionate use starting in 2009 when the current guidelines for mCRC treatment had already been defined. This patient cohort, therefore, had been screened for K/N-ras mutational status and received accordingly at least two poly-chemotherapy lines +/– mAbs to VEGF and/or EGFR prior to receiving the GOLFIG chemo-immunological treatment. Our analysis confirmed the promising antitumor activity of the GOLFIG in mCRC patients, where we recorded a PFS and an OS of 12.55 and 20.28 months, respectively, which was not correlated to age, sex, sidedness, and K/N-ras mutational status in previous treatment lines. The present study confirmed the occurrence of irAEs in 24% of the patients who received the chemo-immunological treatment that was correlated to a longer OS. This finding, together with the previous immunological results reported in the previous study (35–37), strongly supports the hypothesis of an effective immune-response involvement triggered by this chemo-immunotherapy regimen. In line with the literature, the activity of GOLFIG chemo-immunotherapy was explained on the hypothesis that a longer survival mainly depends on the presence of an efficient host's immune response (38) consequent to cytotoxic drugs able to induce immunogenic cell death and autophagy (39) and antigen remodeling and promote immunological danger signals that, in turn, may empower an efficient tumor-specific immune response (40–43). There is additional evidence that cytotoxic drugs such as 5-FU, gemcitabine, or oxaliplatin may selectively kill PDL-1/-2+ immunosuppressive inflammatory cells or Treg cells (26, 31, 34, 38, 40). In line with this evidence, the results of our previous studies showed in these patients GOLFIG treatment-related empowerment of innate immunity, DC-mediated immune-priming, cytotoxic Th1 immune-phenotype, and Tcm cell response paralleled by a progressive decline in immunosuppressive Tregs (31, 34). Altogether, these results were also in line with the occurrence of irAEs and its correlation with a good outcome in these patients. Additionally, this is the only immune-oncological treatment that has shown effective antitumor activity in mCRC; the use of PD-1/PDL-1 blockade mAbs, which have been so successful in other common malignancies, has failed in these patients, excluding those bearing a mismatch repair deficiency (14, 15), even in the presence of tumor-specific CTLs. The reasons for this downfall are not completely known, even though it seems to be correlated with a very immunosuppressive colon cancer micro-environment (14), the same micro-environment that may be significantly modified by the use of the GOLFIG regimen. This hypothesis is supported by the promising results of a recent phase Ib trial where the TSPP vaccine to the tumor-associated thymidylate enzyme showed significant immune-biological and antitumor activity in mCRC patients who received a prior chemo-immunological treatment according to the GOLFIG regimen (44, 45).

In conclusion, our clinical results confirm that the GOLFIG regimen is a competitive regimen for fit mCRC patients who had undergone standard frontline chemotherapy +/– mABs. The new findings derived from the present manuscript are the following: (i) the updated analysis of the GOLFIG-2 trial in chemo-naïve mCRC including 62 censored patients with a 15-year follow-up confirmed that the GOLFIG regimen is superior to FOLFOX alone in terms of PFS with a trend of a prolonged OS; (ii) the present study also demonstrated a significant antitumor activity in largely pre-treated patients, and 7.8% of these patients and none in the FOLFOX arm were alive and free of progression after 10 years; and (iii) both PFS and OS were positively correlated to baseline neutrophil counts and occurrence of irAEs, while sidedness, mutational K/N-ras status, age, sex, and performance status did not correlate. The results of this study maintain an open door on the use of GOLFIG in mCRC patients, which deserves further prospective studies. Additionally, these results warrant the idea of testing a combinatory approach of cytotoxic chemotherapy, immune-stimulating cytokines, and immune-checkpoint blockade.

## Data Availability Statement

The data sets analyzed for this study are opportunely stored and can be provided if requested.

## Ethics Statement

The studies involving human participants were reviewed and approved by Siena University Hospital, Siena, Italy. The patients/participants provided their written informed consent to participate in this study.

## Author Contributions

PC, RG, PTas, PTag, RA, MC, AG, LP, GF, and GD'A wrote the manuscript and designed the study with conceptual contribution. NS, CB, NC, EF, and GR collected and analyzed the clinical data. PP, AN, LR, EM, DC, RMA, and AS performed the statistical analysis of the data. DA, VN, AF, SC, MB, and GT performed the editing of the manuscript and formatted the paper for the journal.

### Conflict of Interest

The authors declare that the research was conducted in the absence of any commercial or financial relationships that could be construed as a potential conflict of interest.
